# Unexpected sound omissions are signaled in human posterior superior temporal gyrus: an intracranial study

**DOI:** 10.1093/cercor/bhad155

**Published:** 2023-06-06

**Authors:** Hohyun Cho, Yvonne M Fonken, Markus Adamek, Richard Jimenez, Jack J Lin, Gerwin Schalk, Robert T Knight, Peter Brunner

**Affiliations:** Department of Neurosurgery, Washington University School of Medicine in Saint Louis, St. Louis, MO 63110, USA; National Center for Adaptive Neurotechnologies, St. Louis, MO 63110, USA; Department of Psychology and the Helen Wills Neuroscience Institute, University of California, Berkeley, Berkeley, CA 94720, USA; TNO Human Factors Research Institute, Soesterberg 3769 DE, Netherlands; Department of Neurosurgery, Washington University School of Medicine in Saint Louis, St. Louis, MO 63110, USA; National Center for Adaptive Neurotechnologies, St. Louis, MO 63110, USA; Department of Psychology and the Helen Wills Neuroscience Institute, University of California, Berkeley, Berkeley, CA 94720, USA; Department of Neurology and Center for Mind and Brain, University of California, Davis, Davis, CA 95618, USA; Frontier Lab for Applied Neurotechnology, Tianqiao and Chrissy Chen Institute, Shanghai 201203, People’s Republic of China; Department of Neurosurgery, Fudan University/Huashan Hospital, Shanghai 200031, People’s Republic of China; Department of Psychology and the Helen Wills Neuroscience Institute, University of California, Berkeley, Berkeley, CA 94720, USA; Department of Neurosurgery, Washington University School of Medicine in Saint Louis, St. Louis, MO 63110, USA; National Center for Adaptive Neurotechnologies, St. Louis, MO 63110, USA; Department of Neurology, Albany Medical College, Albany, NY 12208, USA

**Keywords:** auditory cortex, ECoG, mismatch, prediction, salience

## Abstract

Context modulates sensory neural activations enhancing perceptual and behavioral performance and reducing prediction errors. However, the mechanism of when and where these high-level expectations act on sensory processing is unclear. Here, we isolate the effect of expectation absent of any auditory evoked activity by assessing the response to omitted expected sounds. Electrocorticographic signals were recorded directly from subdural electrode grids placed over the superior temporal gyrus (STG). Subjects listened to a predictable sequence of syllables, with some infrequently omitted. We found high-frequency band activity (HFA, 70–170 Hz) in response to omissions, which overlapped with a posterior subset of auditory-active electrodes in STG. Heard syllables could be distinguishable reliably from STG, but not the identity of the omitted stimulus. Both omission- and target-detection responses were also observed in the prefrontal cortex. We propose that the posterior STG is central for implementing predictions in the auditory environment. HFA omission responses in this region appear to index mismatch-signaling or salience detection processes.

## Introduction

### Expectations influence sensory processing

The notion that the brain uses prior knowledge to make predictions about incoming sensory input has gained considerable traction (Arnal and Giraud 2012; Betti et al. 2021; Friston 2009, 2010; Rimmele et al. 2018; Walsh et al. 2020). The idea is that the brain does not process incoming sensory signals in a purely feedforward manner as previously believed (Serre et al. 2007; Sterzer et al. 2018), but implements cortico-cortical feedback that influences sensory processing in a top-down, hierarchical manner (Rao and Ballard 1999; Lee and Mumford 2003; Leszczyński et al. 2020). Subcortical contributions to predictive coding have also been reported (Carbajal and Malmierca 2018). The advantage of a prediction strategy is improved perception and behavior (Anllo-Vento 1995; Mangun 1995; Casas et al. 2020). On a behavioral level, prior knowledge enhances intelligibility of noisy speech. The underlying mechanism for this effect includes rapid expectation-dependent changes in auditory perceptive field responses (Holdgraf et al. 2016). The evidence of expectations influencing early sensory processing has also been shown in the visual system as reductions of the V1 BOLD response to expected gratings (Alink et al. 2010) and expected tones reduce the auditory N100 amplitude in MEG recordings (Todorovic and de Lange 2012).

### Prediction and the brain

The idea of the brain as a prediction machine was first proposed by Helmholtz (von Helmholtz 1867), and has often been described in a hierarchical Bayesian framework in computational models (Rao and Ballard 1999; Lee and Mumford 2003; Friston 2005; Olshausen et al. 2014; Sterzer et al. 2018; Walsh et al. 2020; Betti et al. 2021). This view describes how predictions and expectations based on prior knowledge aid perception and action. It is proposed that higher-order cortical regions communicate predictions to lower-order regions hierarchically through a multitude of recurrent connections (Clark 2013). One theory of the predictive brain is termed predictive coding (Friston 2005). In the predictive coding framework, local neuronal populations compute errors based on top-down predictions, and these prediction errors are propagated up the hierarchy (bottom-up) to influence subsequent behavior (Rao and Ballard 1999; Friston 2005). According to Friston and Kiebel (2009), the brain operates to “explain-away” expected signals from lower levels of processing, providing an account for reduced responses to expected stimuli. However, a discrepancy in the literature arises when on the one hand, predictions are proposed to reduce neural responses lower in the sensory hierarchy (Friston 2010), yet predictable stimuli are more easily decoded from V1 voxels despite smaller BOLD responses (Kok et al. 2012). This suggests that predictions may not simply reduce overall neural activity in sensory processing areas but perhaps facilitate processing the expected stimulus by enhancing stimulus-specific information (Kok et al. 2012).

### Investigating auditory context processing through omissions

Studies investigating predictions (Todorovic and de Lange 2012; Sanmiguel et al. 2013; Kok et al. 2014; Leonard et al. 2016) often manipulate stimulus predictability or embed stimuli in noise, and the resulting auditory activity is a confluence of bottom-up sensory processing and expectation modulations. Here, we aimed to isolate expectation effects in auditory cortex by examining the neural signals to omissions of expected sounds. Omissions of expected sounds have been shown to elicit ERP responses in EEG ~ 100 ms after the expected sound onset, generated in auditory cortices (Sanmiguel et al. 2013; Bendixen et al. 2014). In the visual domain, omission signals in V1 have been shown to contain stimulus-specific information since the omitted stimulus can be decoded from V1 voxels using fMRI. This has been interpreted as an activation of an expected stimulus template (Kok et al. 2014). In the auditory cortex, omitted speech sounds embedded in words can also be recovered from HFA in superior temporal gyrus (STG). In one study, omitted word sections were replaced by noise, yet HFA reconstructions of the omitted section matched the perceptual experience of the subject (Leonard et al. 2016). Evidence for higher-order information influencing human auditory STG HFA response patterns has also been shown in a study using noisy stimuli that become intelligible in the presence of prior knowledge of what is presented (Holdgraf et al. 2016). HFA has been shown to drive the fMRI BOLD response and correlates with local neural firing and supragranular-layer dendritic inputs, providing a link between different methods (Niessing et al. 2005; Ray and Maunsell 2011; Leszczyński et al. 2020). Here, we utilized the high spatial and temporal resolution of the electrocorticogram (ECoG) to (i) isolate prediction-related HFA responses to omissions in human auditory cortex; (ii) define the spatiotemporal dynamics of these responses; and (iii) determine whether these HFA responses carry stimulus-specific information.

## Materials and methods

### Participants and experimental setup

A total of six subjects (1 female, mean age 46, range between 31 and 69) participated in the current study (see Supplementary Table 1 for further demographic information). All subjects had extensive coverage of the lateral STG. An overview of electrode coverage for S1–S6 can be seen in Supplementary Fig. 1. Subjects were recruited from a patient group with medically refractory epilepsy undergoing neurosurgical treatment, and had subdural electrodes implanted for clinical purposes. These patients were tested during clinical monitoring at the bedside, and typically remained implanted for a duration of 4–10 days. All patients gave their informed consent according to the Declaration of Helsinki, and additional verbal consent was given prior to each testing session. Patients were recruited from Albany Medical Center. Institutional Review Boards from Albany Medical Center and UC Berkeley approved the experimental procedures.

Electrodes were comprised of platinum-iridium and spaced 3–10 mm (PMT Corp.). Electrode placement was determined using pre-operative T1 structural MRI scans and post-operative CT scans, and for analysis across subjects, all locations were projected into the common Talairach space (Talairach and Szikla 1980). All steps were performed within the VERA toolbox (Adamek 2020). Exact timing of the auditory stimuli and behavioral responses was determined by recording their onset using an analog channel of the recording system. All signals were digitized at a sampling rate of 1,200 Hz and recorded using a 256-channel biosignal amplifier (g.HIamp with g.TRIGbox, g.tec, Graz, Austria).

### Experimental task

To enhance stimulus predictability, we played a repetition of the pattern “La-La-Ba La-La-Ga” using syllable stimuli created and shared by the Shannon lab at USC (Shannon et al. 1995). We chose to use syllables as stimuli to ensure robust auditory responses in the STG. We chose “Ba” and “Ga” since these syllables have been previously shown to be decodable from this region (Chang et al. 2010). We used a third syllable, “La,” to create a temporal expectation of the “Ba” or “Ga” to be played. The triplet “La-La-Ba” was alternated with “La-La-Ga,” except in omission or target trials. Whether “Ba” or “Ga” was expected was based on the previous syllable triplet. To ensure that the subject was attentive to the sounds, the subject was instructed to use their thumb contralateral to the ECoG implant to push a button when they perceived the syllable “Ta,” which we randomly introduced in place of the “Ba” or “Ga” as a target stimulus 5% of the time. As the task is repetitive, we chose a target stimulus sounding close to the other stimuli to enhance attention to the triplets and prevent the subject from ignoring the stimuli and simply relying on bottom-up salience for target detection. Finally, the relevant task manipulation was the random omission of either “Ba” or “Ga” on 20% of trials. The syllables lasted 400 ms each, and the ISI within a “La-La-Ba” triplet was fixed to 200 ms, and the ISI between triplets was 200 ms. We recorded between three and six blocks in each subject, with each block including 19 omission trials, 8 target trials, and 68 “Ba” and “Ga” presentations, respectively, lasting about 4 minutes.

Except for S1, we also included a baseline task that was presented before the main experiment. This task was identical to the task described above, except that the third syllable (i.e. “Ba” and “Ga”) was always replaced with an omission. This served as a control to exclude rhythmic-induced effects by the presentation of “La-La.” The BCI2000 software presented the experimental paradigm and recorded the ECoG signals and behavioral responses for further analysis (Schalk et al. 2004).

### Signal preprocessing

ECoG signals were corrected for DC shifts, high-pass filtered using a second-order Butterworth filter at 0.05 Hz, and notch-filtered to remove line noise at 60, 120, and 180 Hz, using the IIR-peak filter in MATLAB. Next, we removed any common noise using a common average reference filter (Liu et al. 2015). To create the common-mode reference, we excluded signals that exhibited an excessive 60 Hz line noise level (i.e. ten times the median absolute deviation).

Timing of stimuli and behavioral responses were extracted from the trigger channel which was recorded simultaneously with the ECoG signals. The onsets of omissions were determined as the expected time of the presentation of “Ba” or “Ga.”

### Signal analysis

We first extracted the HFA band amplitude (70–170 Hz) from the recorded ECoG signals by applying a fourth-order Butterworth band-pass filter, followed by a Hilbert transform. To improve the signal-to-noise ratio of our recordings and to reduce the computational complexity of our subsequent analysis, we down-sampled band-passed signals from 1,200 to 400 Hz using MATLAB “resample” function, which uses a polyphase antialiasing filter to resample the signal at the uniform sample rate. For statistical analysis, we extracted the 500-ms-long response period following each stimulus onset. We used a *t*-test to compare the responses to omission trials to a baseline of 20–0 ms before the stimulus was expected.

To control for multiple comparisons across electrodes, we applied a false discovery rate (FDR) correction considering the number of electrodes and time points we compared in our analysis. For visualizing relative power change, we normalized the response periods to the average spectral power throughout a baseline taken 200–0 ms before stimulus onset.

To determine the effect of the electrode location along the anterior–posterior axis on the response to the stimuli, we localized and visualized electrodes using the VERA and NeuralAct toolboxes (Schalk et al. 2004; Kubanek and Schalk 2015; Adamek 2020). Electrodes were selected based on whether significant HFA increases were observed compared to baseline (with FDR correction, 0–500 ms, *P* < 0.05). Electrodes were grouped into two categories: electrodes with (i) significant omission, and (ii) significant “Ba”/“Ga” HFA increases. This distinction served as an independent variable in a linear mixed-effects model, with the Talairach *y*-coordinate (i.e. the anterior–posterior axis of the Talairach brain) as the dependent variable and the subject as a random effect on the intercept to control for inter-individual differences.

For the peak latency and amplitude analyses, the maximum amplitude and latency were calculated by determining the time of peak HFA within the 500-ms-long response window. Peak latencies and amplitudes were calculated individually for each electrode, “Ba” and “Ga” trials combined, as well as for omissions. The peak HFA amplitude was used as an independent variable in a linear mixed effects model to predict the Talairach *y*-coordinate of the electrode, in addition to the electrode-category-based analysis described above.

For summary statistics, latencies were first averaged across electrodes within subjects and subsequently averaged across subjects. The peak latency was then compared to our null hypothesis (i.e. that omission electrodes are not more delayed than “Ba”/“Ga” electrodes), using a linear mixed effects model with the subject as a random variable.

To determine the effect of the experimental conditions on HFA responses, we applied a generalized linear model (GLM) analysis. In the first step of this analysis, we build a GLM (using the “fitglm” function in MATLAB) as follows:


(1) \begin{equation*} y\sim{x}_1+{x}_2+{x}_3+{x}_4+{x}_5 \end{equation*}

where *y* is the HFA response as the response variable, *x*_1_, *x*_2_, …, *x*_5_ are the experimental conditions (“Ba,” “Ga,” “Omitted Ba,” “Omitted Ga,” and “Ta”) as the predictor variables. Throughout the analysis, we used a normal distribution and linear terms for each predictor. Because the predictor variables are discrete values, we did not use an intercept variable for our GLM analysis. Non-zero coefficients indicate that the experimental condition affected the HFA response at the tested location and time point. We used FDR correction to control for multiple comparisons. Specifically, we tested whether the 95% confidence intervals for the coefficients of the experimental conditions in our GLM spanned across the zero point. For those coefficients where the confidence spanned across the zero point, the associated condition did not affect HFA.

In the second step of our GLM analysis, we tested whether “Ba”/“Ga”-responsive locations distinguish between presented and omitted sounds and whether HFA increases in response to omissions distinguished between omitted “Ga” and “Ba.”

For this step, we calculated a differential score by implementing Fisher’s discriminant scores as follows:


(2) \begin{equation*} \frac{\beta_i-{\beta}_j}{CI_i+{CI}_j} \end{equation*}

using estimated coefficients (β_1_, …, β_5_) from our GLM analysis (i.e. the first step described above) and the confidence intervals (CI_1_, …, CI_5_) to contrast the individual experimental conditions, i.e. “Ba” vs. “Ga,” “Ba vs. ‘Omitted Ba’, and ‘Omitted Ba vs. Omitted Ga”. In this analysis, smaller confidence intervals and bigger differences in estimated coefficients between the two experimental conditions are expected to yield a more significant differential score. An example of the calculated differential score is depicted in Supplementary Fig. 2.

## Results

### Behavioral results

Omissions were embedded in a predictable stream of syllables (“La-La-Ba”; “La-La-Ga”) in a target detection task to ensure attention. Randomly, on 5% of trials, the “Ba” or “Ga” syllable was omitted or replaced by a target syllable (“Ta”), requiring the subject to respond with a button press. The behavioral data in Supplementary Table 2 show that subjects responded to targets with median reaction times ranging between 438 and 643 ms (534 ± 67 ms). The average target hit rate across subjects was 92%, with the lowest individual hit rate at 81%. Subject S3 had higher false alarms (4.6%) than the other subjects (0–0.2%, i.e. button presses to “Ba” or “Ga”).

### High-frequency activity responses in auditory regions to syllables

**Fig. 1:**
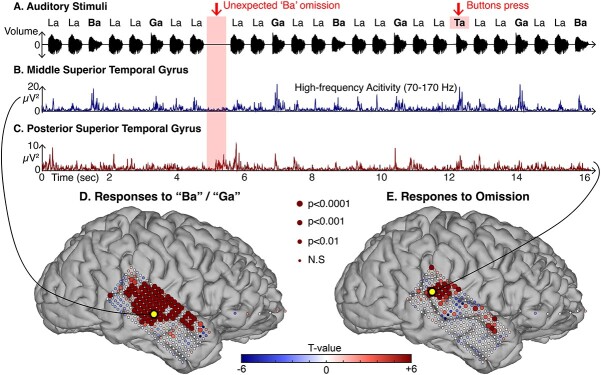
HFA (70–170 Hz) responses for “Ba”/“Ga” and omitted sounds. (A) Examples of the experimental paradigm. Repeated La-La-Ba and La-La-Ga are interspersed with random omissions. (B and C) HFA traces from the middle STG in black and PSTG in red. HFA in PSTG increased for unexpected omissions. (D and E) Topographies of *t*-values by comparing HFA responses in baseline (−200 to 0 ms) and task period (0 to 500 ms) of “Ba”/“Ga” (C) and omitted (D) sounds for all trials of subject S1. The yellow electrode indicates the most significant electrode for each condition (spoken “Ba”/“Ga” or omitted sounds). The size of the electrode corresponds to the significance of the HFA activation from baseline (FDR-corrected).

**Fig. 2:**
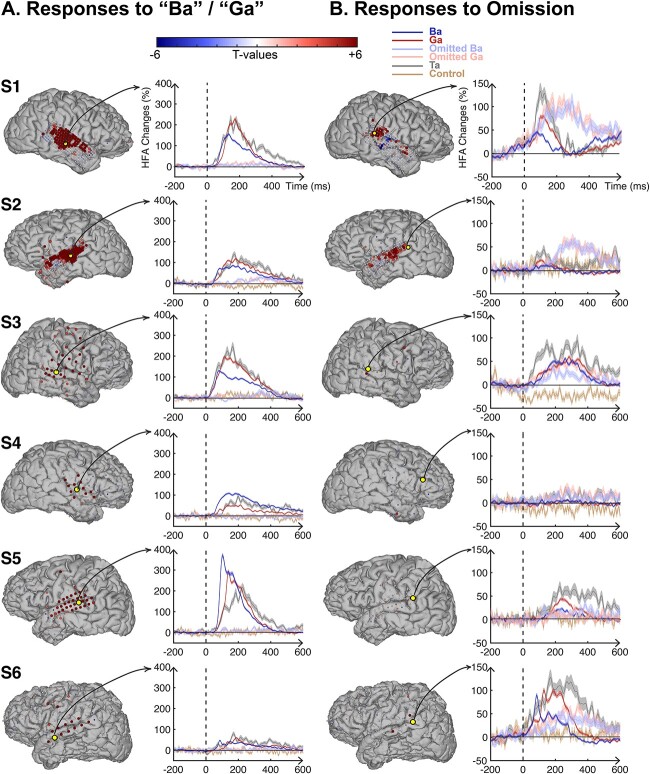
HFA response patterns of spoken “Ba”/“Ga” (A) and omitted (B) sounds for all subjects. Topographies for each individual subject. The color of each electrode corresponds to the amount of task activation (*t*-value of HFA responses relative to baseline (−200 to 0 ms) and task period (0 to 500 ms) of spoken and omitted sounds for all trials). The yellow electrode indicates the most significant electrode for each condition (spoken/omitted sounds). The size of the electrode corresponds the significance of HFA activation from baseline (FDR-corrected). HFA traces are averaged for “Ba,” “Ga,” “omitted Ba,” “omitted Ga” and “ta” presentations. The most significant electrodes showing auditory responses (A) and omission responses (B). Stimulus onset is at 0 ms, and traces are HFA responses to “Ba” (dark blue), “Ga” (dark red), omitted “Ba” (light blue), omitted “Ga” (light red), “ta” (gray), and an omission control (tan, expected omission explained in the Experimental task section of Materials and methods).

Local field potentials were recorded directly from human STG using ECoG grids in six patients undergoing clinical evaluation for medically refractory epilepsy (for electrode coverage, see Supplementary Fig. 1). High-frequency activity (HFA, 70–170 Hz) in response to the auditory stimuli within the temporal lobe for an individual subject (S1) are shown in Fig. 1, with panels (A)–(D) highlighting a subject with a high-density grid (3-mm inter-electrode spacing) covering the STG. In this figure, the size of the electrode represents the significance of the HFA activation relative to baseline (FDR-corrected, *P* < 0.05). “Ba”/“Ga” responses typically occur within 100 ms of stimulus onset, as can be seen in the time courses plotted in panels (A) and (B) (blue and red traces) of Fig. 2. These responses were significant compared to baseline (FDR-corrected, 0–500 ms, *P* < 0.05). The yellow electrode indicates the most significant electrode for each condition (spoken/omitted sounds). All subjects show similar patterns, as can be seen in Fig. 2. Five subjects with posterior STG coverage showed significant omission responses on posterior STG (Fig. 2B, FDR-corrected, 0–500 ms, *P* < 0.05). Subject 4, with sparse posterior STG coverage, did not exhibit an omission response.

### High-frequency activity responses to omissions in auditory regions

Responses to omissions are shown in Fig. 2 (light-colored red and blue traces). HFA increases to omissions predominantly occurred in the posterior part of the STG (see topographies in Fig. 2). Responses to “Ba”/“Ga” syllables in all subjects were found in the middle and anterior STG, with these locations only exhibiting a minimal response to omission (Fig. 2A). In contrast, posterior STG locations still exhibited robust power increases in response to omissions. Only subject S4 showed no omission responses in posterior STG (Fig. 2B). HFA response increases were statistically tested compared to baseline (i.e. 200 ms before the stimulus onset) within subjects using a *t*-test with FDR correction to control for multiple comparisons.

An additional control condition, performed for subjects S2–S6, showed no HFA in response to expected omissions (i.e. “La,” “La,” “Omission”; see Fig. 2). Some locations in STG exhibiting activation in response to omission are unique in that they show both, responses to omissions and targets (“Ta”), but not to “Ba”/“Ga” stimuli (Fig. 2B, the inferior frontal area of S4). No STG omission responses were found in subject S4, which had a limited electrode coverage within posterior STG.

**Fig. 3:**
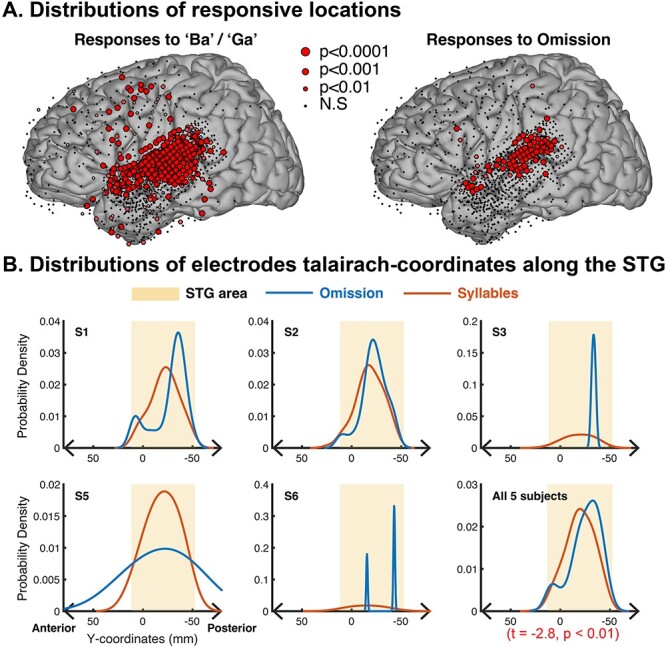
Spatial distribution of auditory responses to syllables and omission. (A) Responses to “Ba”/“Ga” and omission in auditory-active sites, including electrodes from all subjects, projected onto the left hemisphere of a Talairach space. The size of the electrode corresponds to the significance of the HFA activation from baseline (FDR-corrected). (B) Kernel density plots of the Talairach *y*-coordinates along the STG across electrodes for each subject with robust auditory and omission responses. This compares the anterior–posterior location of electrodes with both omission as well as auditory HFA responses (orange) and omission responses (blue). The shaded area indicates the range of STG. Omission responses are more posterior compared with auditory-active electrodes.

To test whether omission-active electrodes were located more posterior on the STG compared to syllable-only active electrodes, we compared Talairach *y*-coordinates (i.e. the anterior–posterior axis of the Talairach brain) of these two groups of electrodes in subjects with extensive STG coverage, including omission responses for subjects S1, S2, S3, S5, and S6. The mean *y*-coordinate difference between omission-active and “Ba”/“Ga”-active electrodes was −11.8 ± 9.7 mm (calculated across subject averages). To determine whether our findings generalize across subjects, we applied a linear mixed effects model to all active “Ba”/“Ga” electrodes, with the subject as a random effect on the intercept. This model found that omission electrodes were more posterior compared to “Ba”/“Ga” electrodes (*P* < 0.01, with a coefficient of −6.7 ± 2.0 (SE) for omission relative to “Ba”/“Ga” electrodes), whereas random effects from subjects were significantly more anterior (a coefficient of 5.6 with a 95% interval of [2.6–11.8]). We also tested whether omission HFA peak amplitude (within a 500-ms-long response time window) could predict the *y*-coordinate of an electrode using a linear mixed effects model. We found that the amplitude of omission HFA again was correlated with the *y*-coordinate (*P* < 0.01, subject as a random effect, *n* = 5 subjects; Fig. 3B). In all five subjects [not including S4, which did not have posterior superior temporal gyrus (PSTG) coverage], sites with omission responses were consistently more posterior than sites responding to “Ba”/“Ga” stimuli, as can be seen in Fig. 3.

**Fig. 4:**
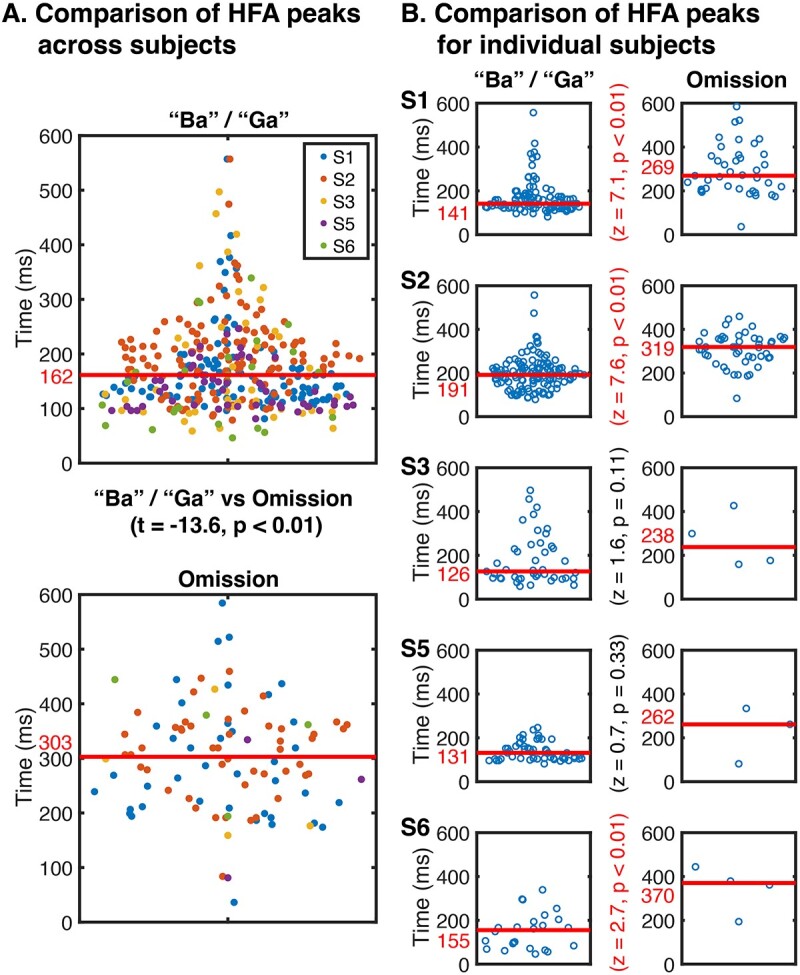
Comparison of HFA peaks from “Ba”/“Ga,” and omission responses (A), in individual subjects (B).

The HFA-responses to omissions exhibited peak latencies that occurred later than those to “Ba”/“Ga”-stimuli. Median omission response peak latencies across subjects ranged between 262 and 370 ms, with a median of 303 ± 77 ms (median absolute deviation, MAD). “Ba”/“Ga” responses in electrodes exhibited peak latencies ranging between 126 and 191 ms, with a median of 161 ± 58 ms (MAD). Across subjects, omission responses peaked between 111 and 225 ms later than “Ba”/“Ga” responses, with a median latency difference of 128 ± 29 ms (median and MAD). A linear mixed-effects model showed omission electrodes were significantly more delayed than “Ba”/“Ga” electrodes (*P* < 0.01, with a coefficient of 121 ± 9.4 ms (SE) for omission relative to “Ba”/“Ga” electrodes). In contrast, the subjects’ random effects coefficient was 17.3 at a 95% confidence within the interval of [7.8 38.6]. This difference between omission and “Ba”/“Ga” latencies could be observed at a single-electrode level (Fig. 2B) and was consistent across subjects (Fig. 4). Overall, omission HFA responses within STG were observed in posterior auditory-active locations and peaked ~ 120 ms later than “Ba”/“Ga” responses.

### GLM analysis using auditory HFA responses to sounds and omissions

**Fig. 5:**
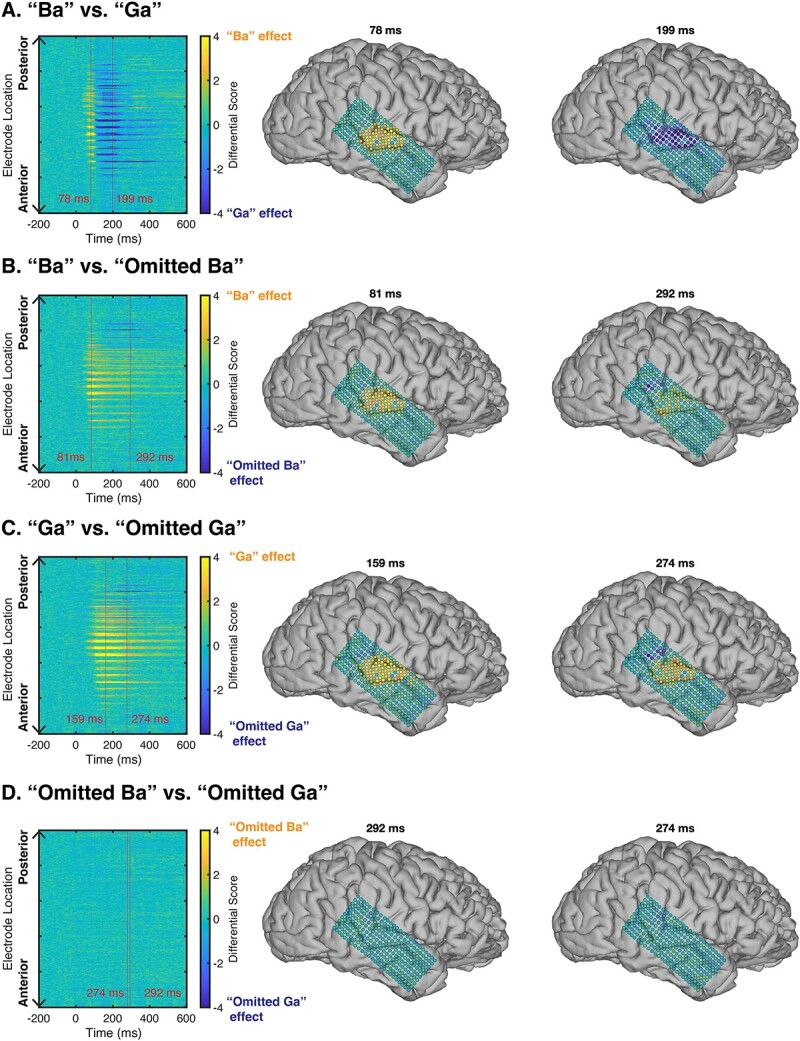
Comparison of the experimental conditions using generalized linear model (GLM) analysis. First, GLM analysis was applied to investigate the effect (beta coefficients) of the experimental conditions (“Ba,” “Ga,” “Omitted Ba” and “Omitted Ga”) to HFA responses. Next, the difference between two effects of different condition was calculated as “Ba” vs. “Ga” (A), “Ba” vs “Omitted Ba” (B), “Ga” vs “Omitted Ga” (C), and “Omitted Ba” vs “Omitted Ga” (D). (A) Topographies are from the specific time points in the channel by time map of differential effects between two conditions. A positive value (yellow color) means that the “Ba” effect was bigger than “Ga” and a negative value (blue color) means that the “Ga” effect was bigger than “Ba” for each electrode. This GLM analysis yields “Ba” and “Ga” features that can be distinguished in a time domain (see the channel by the time map in A), whereas spoken and omitted sounds can be spatially distinguished (see topographies of A and B).

GLM analysis of “Ba” vs. “Ga” showed that the effects of the “Ba” stimulus on HFA responses increased earlier than the effect of “Ga.” As shown in Fig. 5(A), for a representative subject (S1), the spatial pattern of the HFA response to the “Ba” stimulus was similar to that to “Ga” stimulus, but in the time domain, the HFA responses to “Ba” and “Ga” could be distinguished. All subjects consistently exhibited this spatiotemporal pattern (Supplementary Figs. 4–8).

To test whether spoken sound and omitted sound had statistically different spatiotemporal effects on their HFA responses, we compared the HFA responses to “Ba/Ga” and “Omitted Ba/Ga” stimuli, as shown in Fig. 5(B) and (C). In the spatial domain, the effects of spoken and omitted sounds were different. The spatial pattern of HFA response to omitted sounds was mainly observed in posterior STG, with the peak effect occurring later than that for spoken sound (Fig. 5B). In other words, the HFA response to omitted sound was spatiotemporally distinct (posterior STG and delayed latency) from spoken sound (Fig. 5B). As shown in Supplementary Figs. 4–8, the spatiotemporal pattern of the omission response was clear in subjects S1, S2, and S6, but not in subjects S3 and S5. To test whether the failure to see a clear omission response in subjects S3 and S5 may be due to the endogenous, and thus not perfectly time-locked nature of the omission response, we averaged their omission responses across a 500-ms-long window following the stimulus onset. As shown in Fig. 2, following this averaging across the response time, these subjects showed robust posterior STG omission responses.

To test if “Omitted Ba” and “Omitted Ga” elicited different HFA responses in spatial and temporal domains, we applied a GLM analysis similar to that described above to the HFA responses to “Omitted Ba” and “Omitted Ga” stimuli (Fig. 5D). The results of this analysis show there was no distinct difference between the HFA responses to “Omitted Ba” and “Omitted Ga” stimuli (Fig. 5B and Supplementary Figs. 4–8). Therefore, we found that the identity of the omitted stimulus is not encoded in the HFA response to the omission.

### HFA in STG to target stimuli

To investigate the potential presence of misprediction effects, i.e. the cognitive response to “Ba” or “Ga” instead of the target “Ta,” we compared the HFA responses to “Ta” to those of “Ba” and “Ga.” Target sounds elicited an enhanced HFA auditory response (i.e. an onset at 50–150 ms) in some STG electrodes for those five out of six subjects with STG coverage. After the initial evoked response, HFA power remained elevated for target stimuli up to ~ 600 ms post-stimulus (e.g. see Fig. 1B, D, E).

In addition, target stimuli also elicited HFA responses in the prefrontal cortex (see Supplementary Fig. 3). To investigate whether these differences overlapped with omission responses in STG, we determined the ratio of electrodes (activated by the target stimulus) that were also omission-active and omission-silent auditory-responsive electrodes. We did not identify consistent differences between omission-active versus omission-silent auditory electrodes with respect to target stimulus activity.

## Discussion

We examined intracranial recordings to investigate expectation effects on auditory cortex. We isolated the stimulus-independent predictive neural activity by examining omissions of expected sounds and found that a posterior subset of electrodes in the STG robustly responded to omitted sounds as determined by HFA (Figs. 1–Fig. 3). However, information on which stimulus was omitted was not encoded in these HFA power increases (Fig. 5D).

### Posterior STG activates to omissions of expected sounds

We found that HFA increases were elicited by omissions of expected speech sounds. These responses were observed in all subjects except S4, which may be due to limited coverage over posterior auditory areas in STG. A notable finding was that omission responses were observed predominantly in the posterior region of the STG (Fig. 2B). Omission responses were absent in an additional control condition with expected omission, excluding the possibility that the “La-La” sequence rhythmically induced omission responses.

Because we used spoken sounds as stimuli, we could uniquely map out auditory processing regions in STG that may not respond to simpler stimuli, such as tones that are often used in mismatch studies (Edwards et al. 2005; Dürschmid et al. 2016). These results challenge current prediction error signal accounts and theories of sensory cortex-dependent mechanisms of predictive coding. In these models, a prediction error is hypothesized to be produced and propagated along the hierarchy of sensory processing (Friston 2010; Bastos et al. 2012). However, our HFA omission responses were generated in posterior STG, and HFA power to omissions remained at baseline in the majority of STG electrodes that responded strongly to speech sounds.

This anterior versus posterior separation of omission activity in the STG may be related to anatomical separations of the auditory processing stream. A study by Ozker et al. in 2017 shows that noisy speech in the presence of contextual cues differentially affects posterior and anterior STG (Ozker et al. 2017). HFA responses to speech in the posterior STG were unaffected by added noise with context, whereas the word stimuli (“rain” or “rock”) responses degraded in the anterior STG, suggesting an audio-visual integration role for posterior STG. In our task, contextual information arose from knowledge of the stimulus sequence structure, supporting the role of this region in contextual processing. Damage to the posterior STG and angular gyrus has been associated with a specific auditory short-term memory deficit (Markowitsch et al. 1999; Turk et al. 2002). Therefore, the posterior STG may be supporting auditory short-term memory critical for recognizing patterns and signaling deviations.

An anterior–posterior division has also been reported in right STG for consonant compared to dissonant cords processing (Kam et al. 2018). This division has also been reported for spoken sentences, with the posterior STG responding mostly to onsets of sentences and emphasizing syllables. In contrast, the anterior STG remains active throughout the sentence, suggesting a specialization of temporal or salience processing in the posterior STG compared to feature processing in the anterior STG (Hamilton et al. 2018). This is also supported by the view that the posterior and anterior auditory cortex are divided into a dorsal and ventral stream (Bizley and Cohen 2013). In our study, contextual information was present for both when and what sound would follow. A violation of this context activated posterior auditory STG sites, showing that contextual information affects auditory HFA responses differentially following an anterior–posterior division. Combined, these studies and our data point to a specialized role of posterior STG within the auditory stream for the implementation of contextual information.

### Omission responses are temporally persistent

Another characteristic of the HFA increases in omissions is a delayed peak latency compared with syllables responses. The largest omission HFA amplitudes across subjects peaked between 238 and 370 ms. Omission responses in STG peaked ~ 120 ms later compared with “Ba”/“Ga” responses. For one subject, we observed a significant early omission response with a peak onset latency within the first 100 ms of the HFA response. This was most notable in subject S1 (Fig. 2B), in which the posterior electrode shows HFA deviating from baseline as early as 0 ms to both sounds and omissions.

These omission signals may represent two functional roles with differential temporal profiles: (i) responses could signify preparatory processes as part of a predictive process and would be expected to occur early (<100 ms); (ii) omission responses could also signal surprise in the form of a mismatch or prediction error (Wacongne et al. 2011), and/or auditory saliency detection (Downar et al. 2000). Given our results, we may be seeing both processes within the same region. First, HFA may be elevated in anticipation of a stimulus, which subsequently turns into a surprise signal once the expected stimulus fails to appear. Anticipatory neural firing has been observed in the rat auditory cortex in a task that manipulated temporal expectations (Jaramillo and Zador 2011). We may be seeing a neural correlate of this rodent finding in HFA in S1, although this preparatory signal was not consistently observed across subjects.

In contrast, we found robust HFA responses > 100 ms to omissions in all subjects with sufficient STG coverage. These responses peaked ~ 120 ms after the “Ba”/“Ga” -evoked HFA response. This longer latency response may represent a contextual integration process that unfolds after initial sensory processing stages instead of prediction-error signals produced in auditory regions at the expected time of the missing sound. According to predictive coding theory, an unexpectedly omitted sound would produce a prediction error along the auditory processing hierarchy (Friston 2005; Bastos et al. 2012). However, in our data, not all auditory regions display an HFA response to unexpected omissions. The HFA omission response observed may reflect a feedback response from higher-order areas rather than a proactive prediction error. Indeed, the HFA response measured in intracranial EEG has been shown to receive a larger contribution from late supragranular-layer dendritic inputs that likely reflect feedback from other cortico-cortical connections (Leszczyński et al. 2020). The HFA omission response in the posterior STG may reflect such dendritic input from other cortical regions instead of a locally produced prediction error. Such a process may serve to detect saliency, including mismatches between what is expected and actual sensory input. As the posterior STG has been previously implicated with auditory mismatch detection and the ventral attention network (Downar et al. 2001), the HFA omission response is best described as a surprise or mismatch signal.

### Target stimuli elicit increased HFA responses in auditory STG

Target stimuli show larger HFA power increases compared to random, non-target stimuli (“Ba” and “Ga”). Analyses investigating whether such an increase may be larger in “Ba”/“Ga”-active electrodes that also show omission responses did not show consistent effects. The observed increase likely represents a target detection-related attentional modulation (Kastner et al. 1999; Kam et al. 2018), or reflects task-related responses including button press and stimulus–response binding activity as observed in a intracranial study (Haller et al. 2018). It should be noted that contrasting the target “Ta” syllable with non-target “Ba” and “Ga” syllables is not ideal for investigating differences in auditory evoked activity. Syllabic responses can be differentiated in human STG (Chang et al. 2010), with differences attributed to syllabic response in specific electrodes. Moreover, even though the energy in the stimuli was matched, there are differences in spectral properties in the syllable stimuli. Target HFA responses are more reliable in posterior STG/TPJ and inferior frontal cortex than in electrodes with no “Ba”/“Ga” response. Despite the limited coverage of the lateral frontal cortex, our results provided some evidence for overlapping omission and target responses in IFG (Fig. 2B; Supplementary Fig. 3).

### No evidence for stimulus-specific information in omission HFA

We found electrodes in posterior STG encoding syllabic information, evident from different amplitudes and time-courses distinguishing between heard syllables (Fig. 2) and syllable-encoding on HFA responses was robust across STG electrodes (Fig. 5). To test if a stimulus template was activated during omissions of expected sounds, we applied GLM analysis to the HFA time-courses in omission trials. In our analysis, we were unable to predict which syllable was omitted, providing evidence that the identity of the omitted syllable is not encoded in the HFA responses.

Based on previous decoding and encoding approaches, HFA is the most prominent signal containing information on stimulus-specific predictions (Chang et al. 2010; Flinker et al. 2011; Pasley et al. 2012; Martin et al. 2014; Holdgraf et al. 2016). However, it is possible that stimulus-specific expectation information is not carried solely by activity in the HFA. For example, the gamma (30–70 Hz) and beta (15–30 Hz) frequency bands have been previously implicated in prediction processes (Arnal and Giraud 2012; Bastos et al. 2012). To test this, we used the same GLM approach, but with gamma and beta power as predictors trying to decode which stimulus was omitted, which proved unsuccessful.

This discrepancy with other studies finding template activations could be explained by the lack of ambiguity in the present design compared to conditions in a related intracranial study (Leonard et al. 2016), which used distorted phoneme of two different words (e.g. walkers vs. waters.) for the ambiguity. Moreover, such template-specific activations may be most prominent in primary sensory areas (Kok et al. 2012, 2014), which aren’t covered by our lateral temporal surface electrodes. Finally, it is possible that the omission of a sound is more salient than the omission of a syllable. We speculate that the omission response observed here may be related to temporal violations, saliency detection, or both.

### Contextual processing and the posterior STG

A large body of work assigns a multitude of functions to the posterior STG, including speech processing, face processing, audiovisual integration, motion processing, and theory of mind (Hein and Knight 2008). This region is also linked to the ventral attention network, which comprises posterior STG, the temporoparietal junction (TPJ), inferior frontal gyrus (IFG), insula, and cingulate cortex (Downar et al. 2002). This network is linked to identifying salient events and to re-orientation of attention (Corbetta and Shulman 2002; Downar et al. 2002). This is not surprising, as a deviation from the expectation of what is coming next is core to classifying a stimulus as novel and potentially salient. Contributions from the TPJ and its role in the generation of the P300 ERP have been linked to contextual updating (Geng and Vossel 2013). Contextual updating would update the prediction for the next trial based on the outcome of the current trial. The full cycle of this prediction process follows a time course that extends beyond early sensory processing. Our data provide insight into the recruitment of auditory regions and their temporal dynamics at different stages of this process. Similar to a previous study (Downar et al. 2001), we found omission response in the posterior STG (Fig. 5). Some anterior and posterior STG sites were involved specifically in “Ba”/“Ga” and omission processing, whereas some more posterior sites did not show “Ba”/“Ga” responses and responded more strongly to targets over omissions (Fig. 2B). This is in accord with modality-general TPJ responses, whereas the posterior STG is specific to auditory novelty (Downar et al. 2002). The posterior STG may comprise the first node in the network for the detection and response to salient auditory events. In addition, limited sites in IFG were also active to omissions (Figs. 1–Fig. 2). Given the apparent non-specific, prolonged nature of the HFA omission response, local neural activity underlying this response might be involved in binding anticipatory processes with the auditory mismatch processes and the salience detection network.

## Conclusions

We show that omissions of expected sounds elicit a robust HFA increase in the posterior STG electrodes following a division of anterior vs. posterior auditory activity in STG (Ozker et al. 2017; Hamilton et al. 2018). In contrast to the current prediction theories, a GLM analysis applied to this HFA increase in the STG was unsuccessful in distinguishing which stimulus was omitted, suggesting that the observed omission of HFA response does not carry stimulus-specific information. Finally, this response is different from that seen in the TPJ, which was shown to respond to both omissions and targets, but not to sounds generally.

## Supplementary Material

Supplementary_materials_bhad155

syllable_Ba_bhad155

syllable_Ga_bhad155

syllable_La_bhad155

syllable_Ta_bhad155

## Data Availability

Data are available from the corresponding author Peter Brunner (pbrunner@wustl.edu) upon request.
