# Supplementary material for: Unexpected sound omissions are signaled in human posterior superior temporal gyrus: an intracranial study
Source: Cereb Cortex. 2023 Jun 6;33(14):8837–48. doi: 10.1093/cercor/bhad155 (PMC10350817; doi:10.1093/cercor/bhad155)
Supplement: Supplementary_materials_bhad155 [file supplementary_materials_bhad155.pdf]

Supplementary Table 1 Subject demographic information. * denotes unknown.

Subject	*Gender*	*Age*	*Handedness*	*Hand used*	*Language lateralization*	*Seizure focus*
S1	F	33	Right	Left	*	*
S2	M	33	Right	Right	Left	Anterior depth
S3	M	57	Left	Left	Left	*
S4	M	69	Right	Left	*	Anterior ventro-temporal
S5	M	31	Right	Right	*	Ventral/temporal pole
S6	M	51	Right	Right	*	Left frontal

Supplementary Table 2 Behavioral data per subject. Reaction times to targets in milliseconds, hit rate in percentage, misses and false alarms in number of trials total.

Subjects	*RT median (ms)*	*RT mean (ms)*	*RT standard deviation (ms)*	*Hit rate*
S1	643	661	224	98%
S2	527	563	193	85%
S3	438	481	160	81%
S4	550	578	147	92%
S5	500	528	122	96%
S6	548	547	103	100%
Average	**534**	**560**	**158**	**92%**

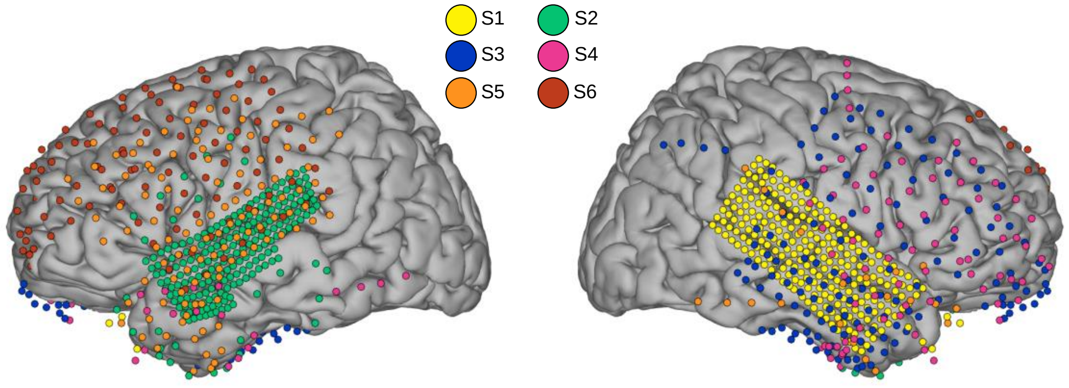

Supplementary Figure 1 Overview of electrode coverage across subjects in Talairach space. Color signifies an individual subject.

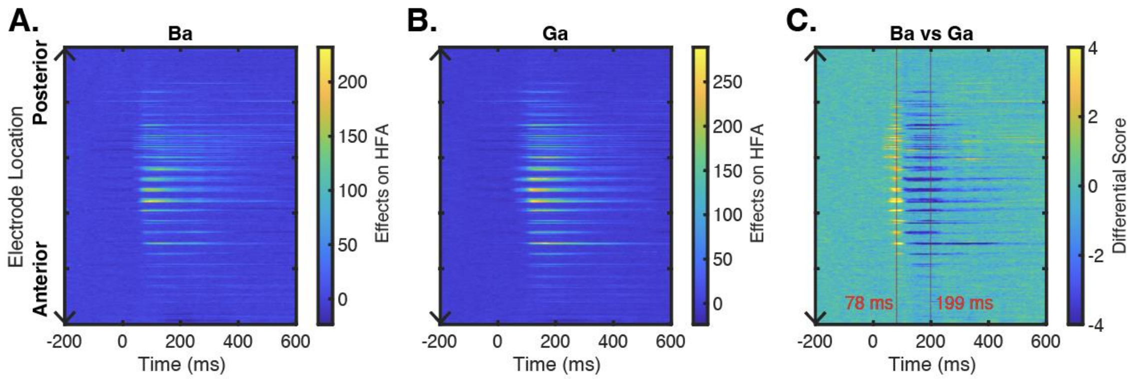

Supplementary Figure 2 An example of estimated coefficient map from experimental condition Ba (**A**) and Ga (**B**), differential score map for Ba vs. Ga (**C**).

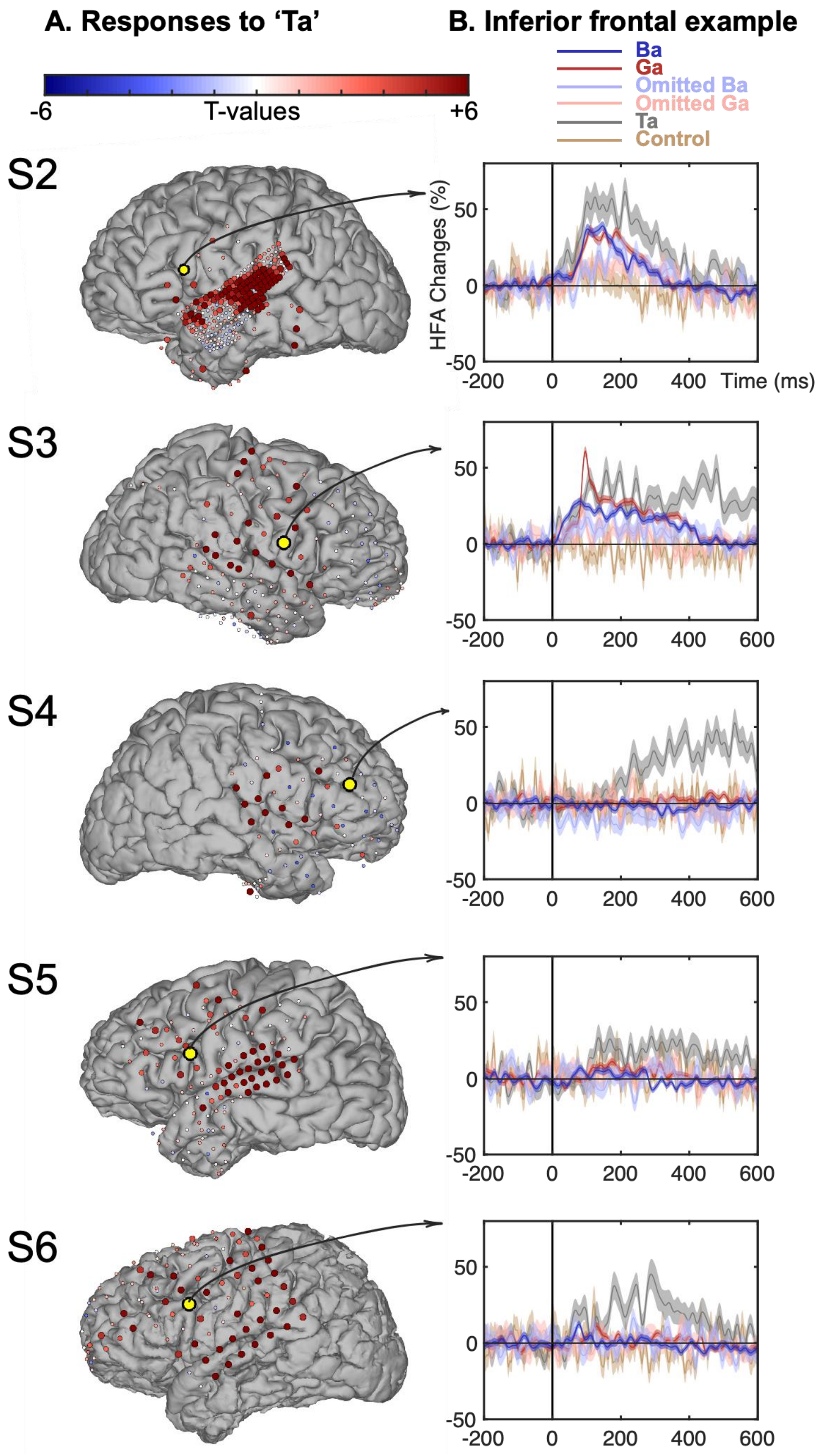

Supplementary Figure 3 Target ('Ta') HFA power increases in inferior frontal cortex. **A.** Topography of significant auditory HFA power change from baseline period (-200 to 0 ms). **B.** Example auditory electrodes (yellow electrode in the topography) in inferior frontal cortex. Stimulus onset is at 0ms, and traces are HFA responses to 'Ba' (dark blue), 'Ga' (dark red), omitted 'Ba' (light blue), omitted 'Ga' (light red), 'Ta' (gray) and an omission control (brown).

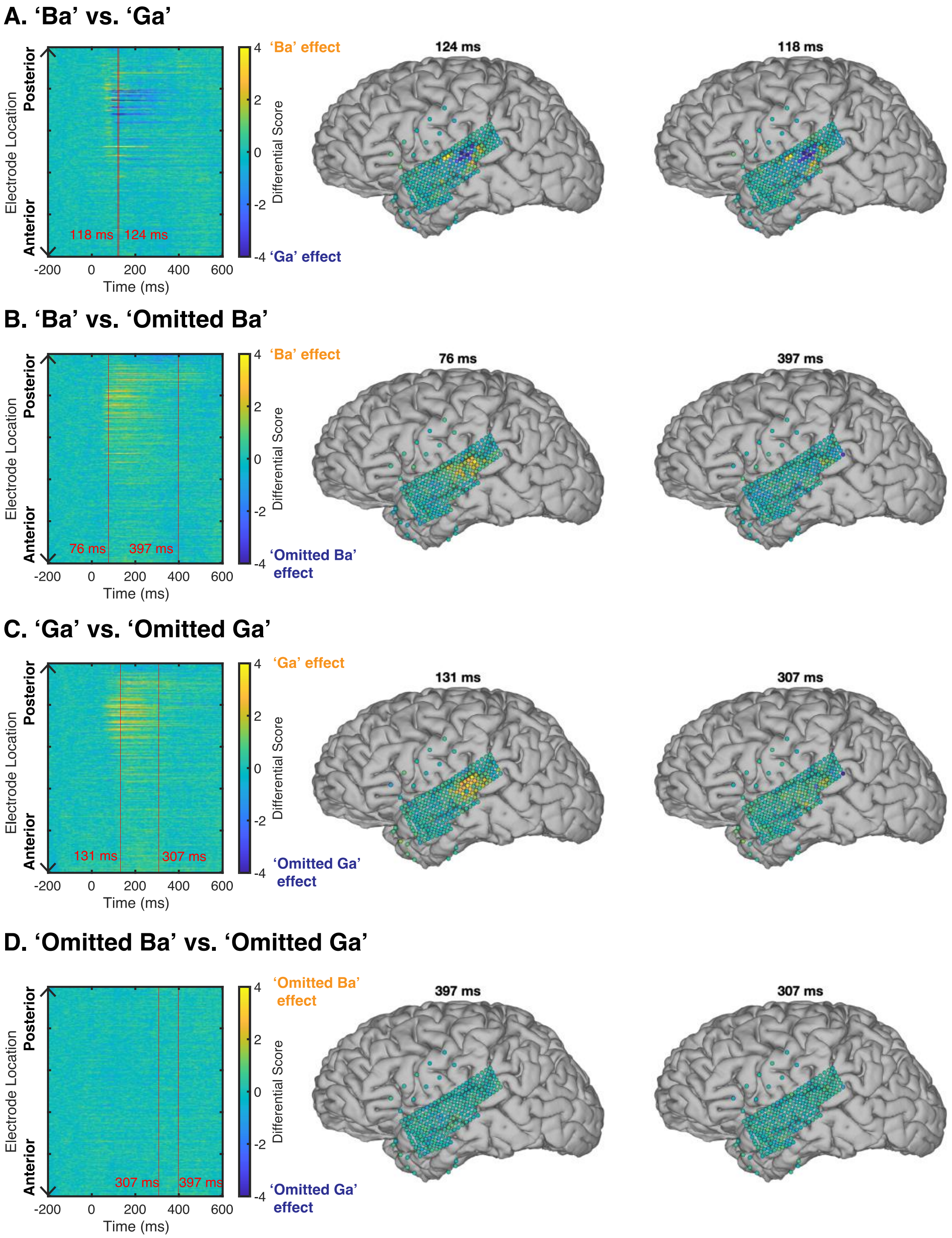

Supplementary Figure 4 A-D. Comparison between experimental conditions using GLM analysis (subject S2).

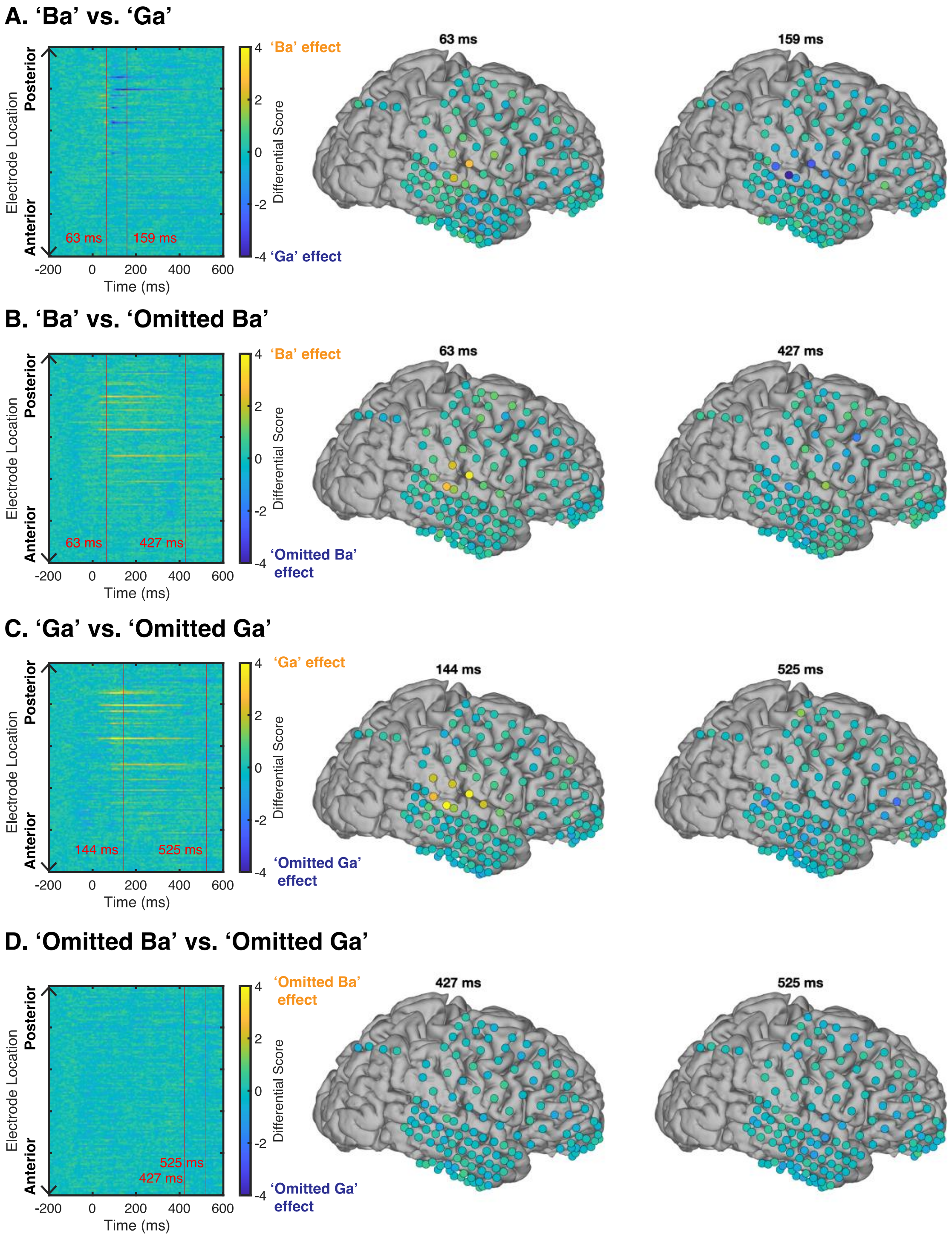

Supplementary Figure 5 A-D. Comparison of the experimental conditions using GLM analysis (subject S3).

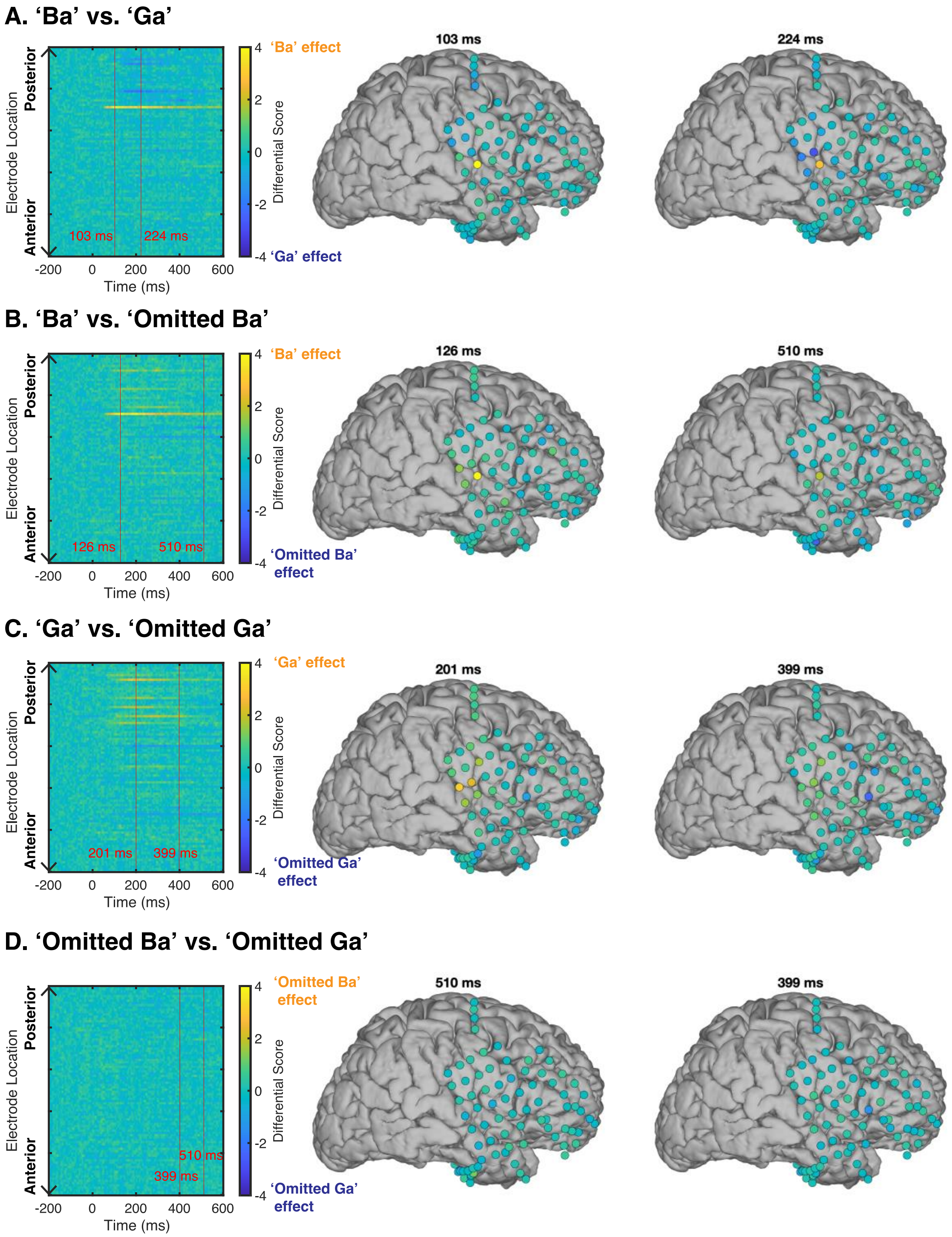

Supplementary Figure 6 A-D. Comparison of the experimental conditions using GLM analysis (subject S4).

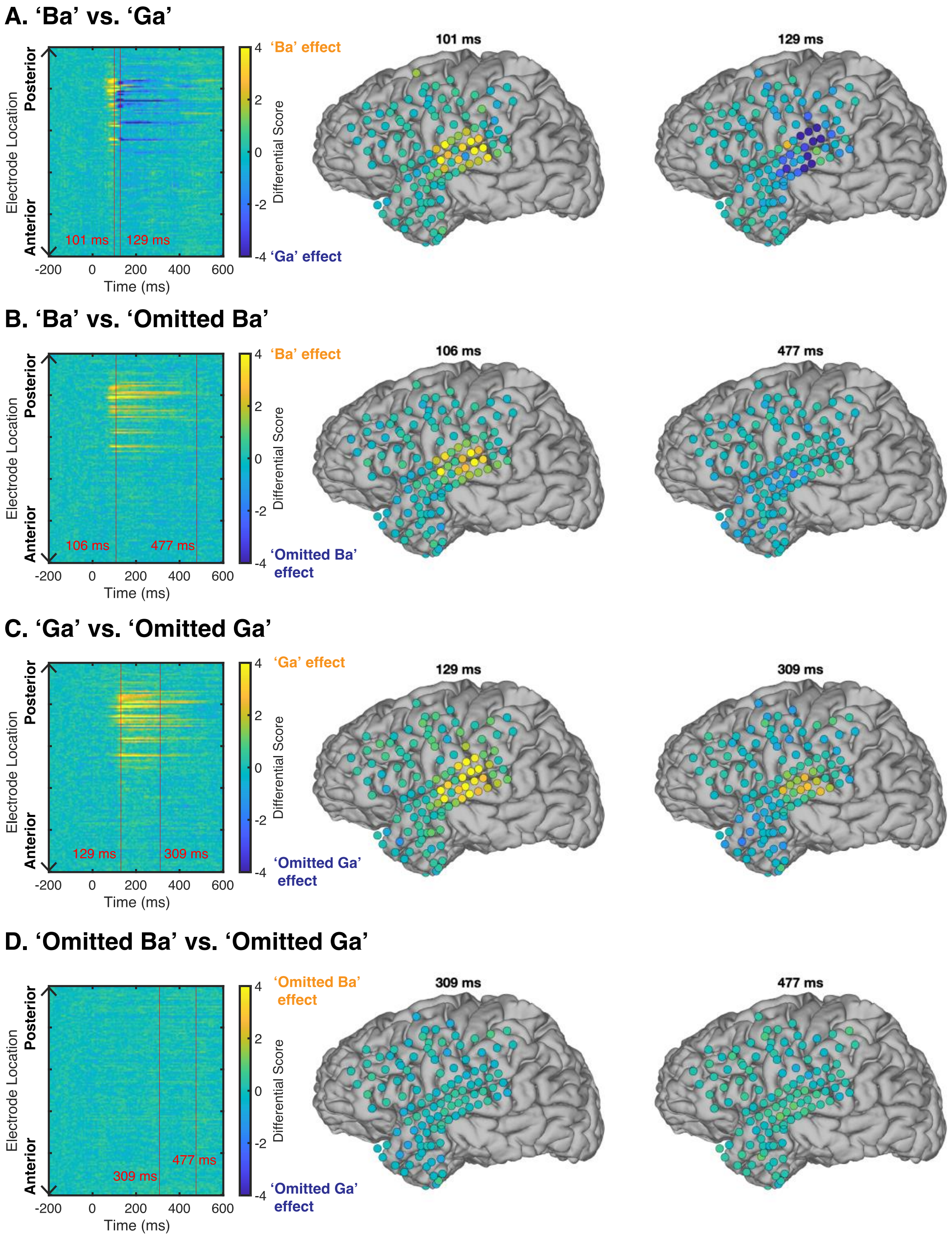

Supplementary Figure 7 A-D. Comparison of the experimental conditions using GLM analysis (subject S5).

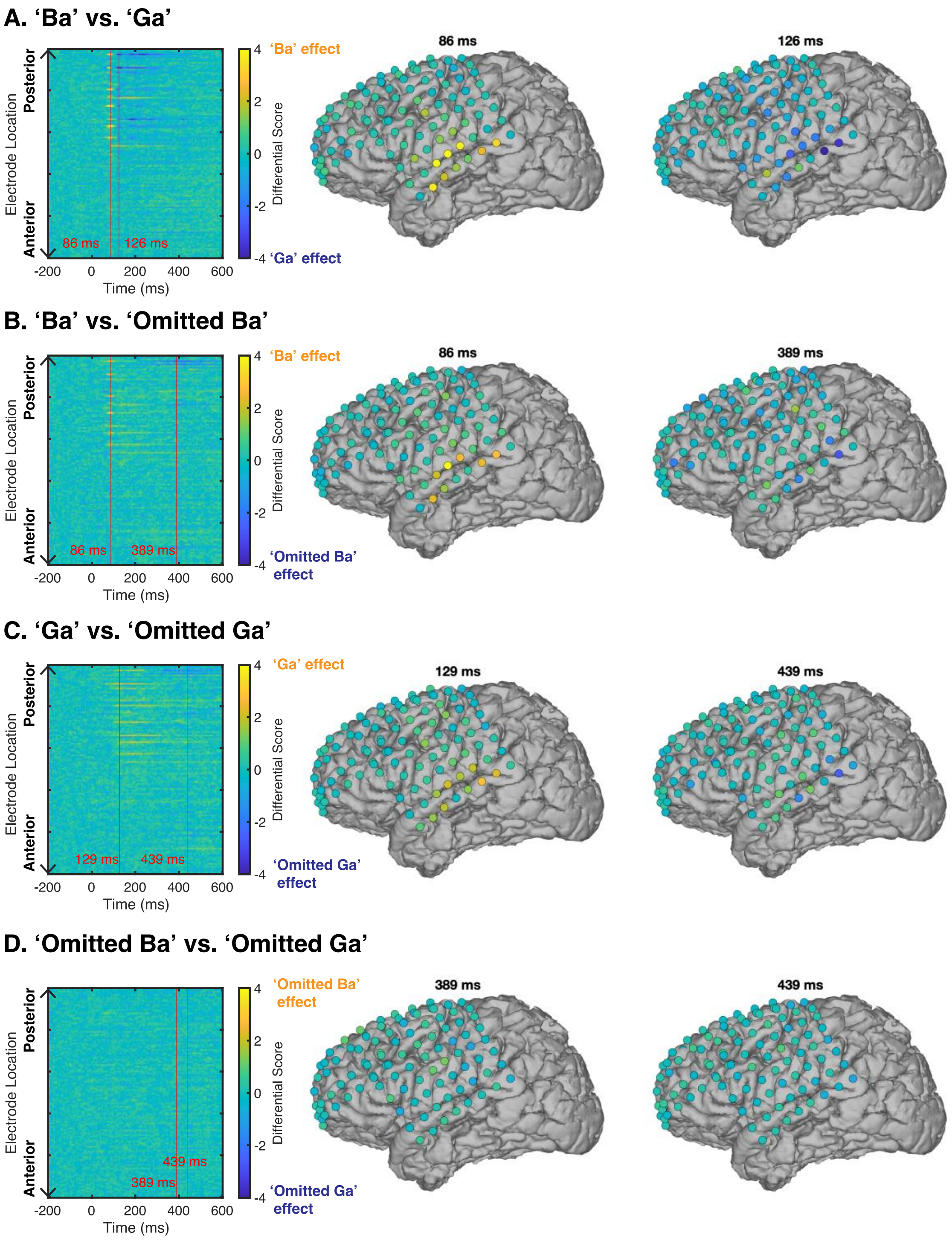

Supplementary Figure 8 A-D. Comparison of the experimental conditions using GLM analysis (subject S6).
